# Drug resistance occurred in a newly characterized preclinical model of lung cancer brain metastasis

**DOI:** 10.1186/s12885-020-06808-2

**Published:** 2020-04-07

**Authors:** Neal Shah, Zhongwei Liu, Rachel M. Tallman, Afroz Mohammad, Samuel A. Sprowls, Pushkar A. Saralkar, Schuyler D. Vickers, Mark V. Pinti, Weimin Gao, Paul R. Lockman

**Affiliations:** 1 Department of Basic Pharmaceutical Sciences, School of Pharmacy, West Virginia University, 108 Biomedical Drive, Morgantown, WV 26506 USA; 2 School of Medicine, West Virginia University, 1 Medical Center Drive, Morgantown, WV 26506 USA; 3 Department of Occupational and Environmental Health Sciences, School of Public Health, West Virginia University, 64 Medical Center Drive, Morgantown, WV 26506 USA; 4 Department of Pharmaceutics, College of Pharmacy, University of Minnesota, Minneapolis, MN 55455 USA

**Keywords:** PC-9, Brain metastasis, Drug resistance, EGFR-TKI

## Abstract

**Background:**

Cancer metastasis and drug resistance have traditionally been studied separately, though these two lethal pathological phenomena almost always occur concurrently. Brain metastasis occurs in a large proportion of lung cancer patients (~ 30%). Once diagnosed, patients have a poor prognosis surviving typically less than 1 year due to lack of treatment efficacy.

**Methods:**

Human metastatic lung cancer cells (PC-9-Br) were injected into the left cardiac ventricle of female athymic nude mice. Brain lesions were allowed to grow for 21 days, animals were then randomized into treatment groups and treated until presentation of neurological symptoms or when moribund. Prior to tissue collection mice were injected with Oregon Green and ^14^C-Aminoisobutyric acid followed by an indocyanine green vascular washout. Tracer accumulation was determined by quantitative fluorescent microscopy and quantitative autoradiography. Survival was tracked and tumor burden was monitored via bioluminescent imaging. Extent of mutation differences and acquired resistance was measured in-vitro through half-maximal inhibitory assays and qRT-PCR analysis.

**Results:**

A PC-9 brain seeking line (PC-9-Br) was established. Mice inoculated with PC-9-Br resulted in a decreased survival time compared with mice inoculated with parental PC-9. Non-targeted chemotherapy with cisplatin and etoposide (51.5 days) significantly prolonged survival of PC-9-Br brain metastases in mice compared to vehicle control (42 days) or cisplatin and pemetrexed (45 days). Further in-vivo imaging showed greater tumor vasculature in mice treated with cisplatin and etoposide compared to non-tumor regions, which was not observed in mice treated with vehicle or cisplatin and pemetrexed. More importantly, PC-9-Br showed significant resistance to gefitinib by in-vitro MTT assays (IC50 > 2.5 μM at 48 h and 0.1 μM at 72 h) compared with parental PC-9 (IC50: 0.75 μM at 48 h and 0.027 μM at 72 h). Further studies on the molecular mechanisms of gefitinib resistance revealed that EGFR and phospho-EGFR were significantly decreased in PC-9-Br compared with PC-9. Expression of E-cadherin and vimentin did not show EMT in PC-9-Br compared with parental PC-9, and PC-9-Br had neither a T790M mutation nor amplifications of MET and HER2 compared with parental PC-9.

**Conclusion:**

Our study demonstrated that brain metastases of lung cancer cells may independently prompt drug resistance without drug treatment.

## Background

Lung cancer is the second-most commonly diagnosed cancer in the United States, and is the most common cause of cancer death worldwide [1, 2]. It is estimated that more than 200,000 new cases of lung and bronchus cancer will be diagnosed and more than 140,000 cancer deaths will occur in the United States in 2019 [2]. The average age of diagnosis is 70, while the median age of death is 72. The short time from diagnosis to death may be due to the advanced stage on presentation [3]. The two most common types of lung cancer brain metastasis (LCBM) are small-cell and non-small-cell lung cancer, the latter having three prominent mutations: KRAS, epidermal growth factor receptor (EGFR), and EML4-ALK. Approximately 85% of lung cancer are non-small cell lung carcinoma (NSCLC) with small-cell lung carcinoma (SCLC) comprising the rest [4]. Adenocarcinoma, the most common subtype of NSCLC, presents with brain metastases in 10% of patients, forming in approximately 40% patients throughout illness progression [3]. Within adenocarcinoma, the most common mutation is KRAS, followed by EGFR and EML4-ALK translocation. Targetable drugs exist for EGFR and EML4-ALK, but not for KRAS. Within the scope of EGFR, the deletion on exon 19 confers sensitivity to targeted inhibitors.

Overall, lung cancer metastasizes to brain in approximately 10 to 30% of patients and is responsible for the majority of brain metastases [5], which is often a fatal prognosis due to a lack of curative treatment modalities [6]. There is no one universal effective screening tool for lung cancer as there are for other cancer types, such as breast cancer or melanoma [7]. Therapeutic options in the treatment of LCBM include surgical resection, stereotactic radiosurgery, whole brain radiotherapy, and chemotherapy [6]. Even when used in combination, these options rarely improve survival beyond 12 months [8]. The presence of the blood-brain barrier (BBB) and blood-tumor barrier (BTB) can significantly hinder penetration of chemotherapeutic agents into both tumor and brain tissues [9]. The BBB consists of a physical barrier of vascular endothelial cells linked together by tight junctions, enzymes such as phosphatases to degrade substances, and efflux transports actively restricting molecular entry into the brain, all surrounded by astrocytic foot processes performing similar activities [10]. In the BTB, immature vasculature structure leads to increased permeability and though drug permeation is enhanced, the magnitude of enhancement often falls below therapeutic amounts required for efficacy [11].

In the current study, we compared tumor progression and survival in a mouse model of LCBM injected with PC-9 (a human lung adenocarcinoma cell line) or PC-9-Br (a newly developed brain-seeking lung cancer cell line). We also evaluated functionality of the tumor vasculature in our model with a passive permeability marker ^14^C-aminoisobutyric acid (^14^C-AIB, MW = 103.12) and a P-glycoprotein (P-gp) substrate Oregon Green (OG, MW = 509.38), as well as albumin-bound vascularity marker indocyanine green (IR-820, ICG). We then shifted focus to treatment and as such mice bearing brain lesions were treated with the clinical combinations of cisplatin+etoposide or cisplatin+pemetrexed. Since PC-9 harboring the deletion mutation on EGFR exon 19 is highly sensitive to EGFR-tyrosine kinase inhibitors (EGFR-TKIs) [12], the sensitivity of PC-9-Br to first-generation EGFR-TKI gefitinib was evaluated in vitro compared with PC-9 parental in this study. The molecular mechanisms of gefitinib resistance were also investigated in this study.

## Methods

### Cell culture

The parental PC-9 cells (EGFR exon19 E746–A750 deletion) were provided by Dr. Lori Hazlehurst’s laboratory, and came transduced to display Tomato Red and Firefly luciferase (Luc2 = tdT), allowing for fluorescence quantification and bioluminescence tracking. The pcDNA3.1(+)/Luc2 = tdT was a gift from Christopher Contag (addgene plasmid # 32904). Cells were grown in RPMI supplemented with 10% fetal bovine serum, 1% penicillin-streptomycin, and 10 μL/mL of G418 to ensure selection of transduced cells. Cells were kept at 37 °C and 5% CO_2_. All cells used for in vivo and in vitro experiments were between passages 1–10.

### Animals and brain tumor model development

Female athymic nu/nu mice (~ 25 g) were purchased from Charles River Laboratories (Wilmington, MA). All animals were aged approximately 6–8 weeks on time of model initiation. Mice were anesthetized using 2% isoflurane. After placement into a stereotactic device (Stoelting), approximately 150,000 of PC-9 cells in 100 μL of PBS were injected into the left cardiac ventricle. Bioluminescence was used to verify presence of PC-9 cells in the brain. Upon termination, animals were euthanized and brains were extracted to begin ex-vivo creation of the PC-9 brain seeking line (PC-9-Br). The protocol developed by Yoneda et al. [13] was similarly followed to establish the PC-9-Br line. Tumor-bearing brains were extracted, partially homogenized, and digested in a collegenase solution in DMEM. The preparation was then extruded through a 19G needle and strained with a 70 μm cell strainer. The preparation was then centrifuged multiple times, following addition of DMEM and FBS, PBS, and 25% BSA in PBS, respectively. The pellet was collected and cultured in media containing G418 to select for transfected cells. After cells had sufficiently proliferated, they were washed with PBS and re-plated for at least 24 h prior to re-injection in mice. This process was repeated until the extracted population predominantly formed intracranial lesions, which was 6 times for the PC-9 line, named as PC-9-Br.

### Longitudinal bioluminescence and survival model

To demonstrate the high morbidity and progression associated with LCBM, we monitored the survival and bioluminescence (BLI) signal after injection of 150,000 PC-9-Br and PC-9 parental cells. Animals were given an intraperitoneal 150 mg/kg injection of d-luciferin potassium salt and anesthetized with 2% isoflurane. Based on the results from unpublished preliminary work, after 10 min of circulation, animals were transferred to the IVIS Spectra CT (PerkinElmer) and BLI was captured at auto-exposure and one-minute time spans on Stage D with medium binning, fitting within the optimal imaging time for the PC-9-Br line. For quantification, a region of interest (ROI) was drawn based on cranial circumference. BLI based on ROI is reported as radiance (photons/sec/cm2/steridian). These mice were monitored regularly for survival until all the mice in PC-9 parental expired. The time and number of deaths in PC-9-Br and PC-9 parental groups were recorded regularly. The experiment was performed under the strict compliance of IACUC of West Virginia University. Data was plotted on a Kaplan Meier curve, which was used to analyze the survival pattern of mice in PC-9 parental and PC-9-Br groups. Mice were euthanized via exanguination under deep ketamine/xylazine (100 mg/kg and 8 mg/kg, respectively) anesthesia.

### Chemotherapy preparation and administration

On day 21, mice were randomized into treatment or vehicle groups and began treatment. Cisplatin (5 mg/kg, weekly) and either etoposide (100 mg/kg, days 2 through 5 after cisplatin administration) or pemetrexed (100 mg/kg, days 3 through 5 after cisplatin administration) were selected to represent the most common nonspecific platinum doublet therapy given to lung cancer patients. Cisplatin and pemetrexed were dissolved in saline, and etoposide was dissolved in 5% DMSO, 5% Tween 80, and 90% saline prior to intravenous dosing. All chemotherapy was purchased from SelleckChem. BLI was taken twice weekly to measure chemotherapy response and tumor burden, performed at least an hour prior to drug administration to avoid interactions.

### Brain extraction, tissue processing, and quantification

Upon reaching survival endpoints, mice were anesthetized and given tail vein injections of 150 μg of OG dissolved in PBS, along with 10 μCi of ^14^C-AIB. Following a 10-min circulation, the descending aorta and inferior vena cava were clamped off. A solution of 6 mg of ICG bound to 0.27% bovine serum albumin (270 mg in 10 mL) was perfused through the left ventricle at 5 mL/min to provide a washout. Brains were then rapidly removed and flash-frozen in isopentane (− 80 °C) and stored at − 80 °C prior to tissue slicing and visualization.

Brains were mounted and 20 μm slices were created with the Leica CM3050S cryotome (Leica Microsystems, Wetzlar, Germany), which were transferred to charged microscope slides. Each slide contains 3 slices for a total of approximately 120 slices per brain. Brain slice fluorescence was acquired using a stereomicroscope (Olympus MVX10; Olympus, Center Valley, PA) equipped with a 0.5 NA 2X objective and a monochromatic cooled CCD scientific camera (Retiga 4000R, QIMaging, Surrey, BC, Canada). Tomato Red fluorescence was imaged using a DsRed sputter filter (excitation/band λ 545/25 nm, emission/band λ 605/70 nm and dichromatic mirror at λ 565 nm) (Chroma Technologies, Bellows Falls, VT), OG using an ET-GFP sputter filter (excitation/band λ 470/40 nm, emission/band λ 525/50 nm and dichromatic mirror at λ 495 nm) (Chroma Technologies, Bellows Falls, VT), and ICG using a Cy7 sputter filter (excitation/band λ 710/75 nm, emission/band λ 810/90 nm and dichromatic mirror at λ 760 nm) (Chroma Technologies, Bellows Falls, VT). Fluorescence was captured and analyzed using CellSens (Olympus) software. OG intensity increases were determined by sum intensity per unit of metastatic lesion area relative to non-tumor brain regions.

### Quantitative autoradiography

Fluorescence imaging slides and ^14^C-AIB slides were placed in quantitative autoradiography (QAR) cassettes (FujiFilm Life Sciences, Stanford, CT) along with ^14^C autoradiographic standards (American Radiochemicals, St. Louis, MO). A phosphor screen (FujiFilm Life Sciences, 20 × 40 super-resolution) was placed with the slides and standards and allowed to develop for 21 days. QAR phosphor screens were developed in a high-resolution phosphor-imager (GE Typhoon FLA 7000, Uppsala, Sweden) and converted to digital images, which were then calibrated to ^14^C standards and analyzed using MCID Analysis software (InterFocus Imaging LTD, Linton, Cambridge, England). Metastases permeability fold-changes were calculated based on ^14^C-AIB signal intensity within confirmed metastases locations (determined using cresyl violet and Tomato Red fluorescence intensity overlays) relative to non-tumor brain ^14^C-AIB signal intensity.

### Tumor staining

Tissue sections were processed as described above. After allowing tissues to become adherent to charged slides overnight, slides were briefly dipped in PBS. Staining was performed using 0.1% cresyl violet acetate (Sigma-Aldrich, St. Louis, MO) (2 min) followed by briefly rinsing in tap water. Sections were cleared in 70% ethanol (15 s), 95% ethanol (30 s), 100% ethanol (30 s), respectively. Images were obtained with a 2× objective on the Olympus MVX microscope.

### Cell viability assay

Cell viability was evaluated by the MTT assay as described previously [14, 15]. PC-9 parental and PC-9-Br were treated by gefitinib at different concentrations for 48 and/or 72 h. Experiments were repeated independently three times.

### Western blot analyses, PCR, and T790M mutation analyses

Protein expressions in PC-9 parental and PC-9-Br were analyzed by Western blot as previously described [14, 15]. α-tubulin was used as an internal control.

Genomic DNAs from PC-9 parental and PC-9-Br were isolated using a DNeasy Blood & Tissue Kit (Qiagen, Valencia, CA, USA). EGFR exon 20 were amplified by PCR according to the method established previously [16]. The PCR products were purified by QIAquick PCR Purification Kit (Qiagen, Hilden, Germany) and sequenced as described in our previous study [15]. For MET, METFR (endogenous control for MET), HER2, and EFTUD2 (endogenous control for HER2), 75 ng of genomic DNA was amplified using SYBR Green Supermix (BioRad). Experiment was performed in triplicate for each group. The PCR primer sequences were reported in the previous studies [14–16].

Total RNA was isolated from PC-9 parental and PC-9-Br using the RNeasy Plus Mini Kit (Qiagen) following the manufacturer protocol. One-step RT-PCR Kit with SYBR green was used for amplification of total mRNA (75 ng) following the manufacturer’s protocol (BioRad, Hercules, CA, USA) and our previous studies [14, 15]. Experiment was performed in triplicate for each group. The PCR primer sequences were reported in the previous studies [14–16].

### Statistics

All statistics were performed on GraphPad Prism software. XY plots were analyzed by linear regression. Median and interquartile ranges are used for permeability changes and size of metastases. A D’Agostino and Pearson omnibus test was performed and determined a non-Gaussian distribution of data. Statistical analysis of permeability and size was performed using the non-parametric Kruskal-Wallis test followed by Dunn’s multiple comparison test. On survival endpoints, mice were sacrificed and date of death recorded. Kaplan-Meier curves were generated and compared using log-rank statistics. Prism was used for calculation of the 50% inhibitory concentrations (IC_50_s). Student’s t test and one-way ANOVA followed by a Fisher’s LSD test were applied to determine the difference in the results of cell viabilities and qRT-PCR. Significance for all tests was defined as *p* < 0.05.

## Results

### The sixth round of PC-9 injections predominantly seeds the brain and has shorter survival than the parental line

**Fig. 1:**
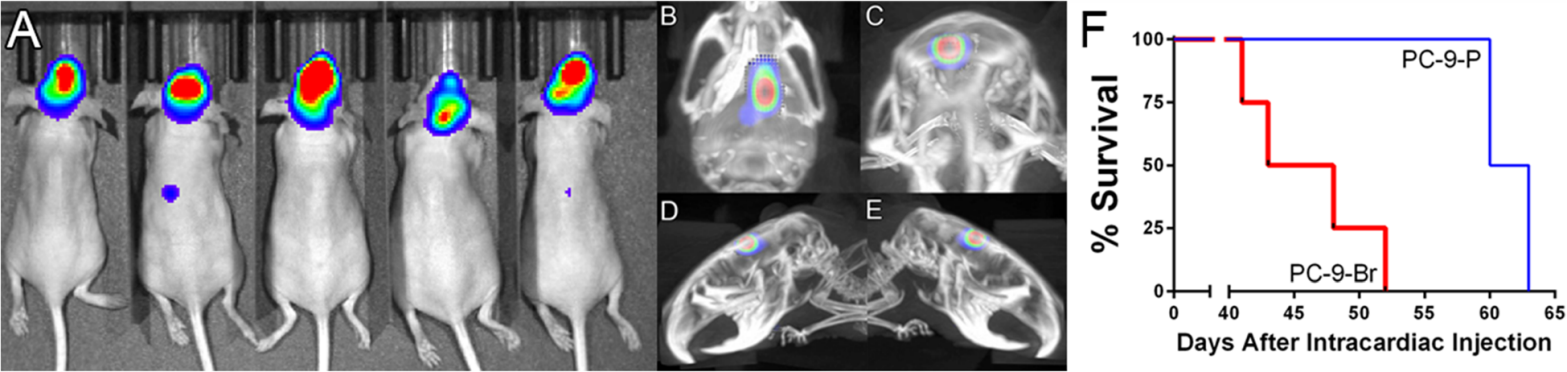
**a** Visualization of tumor burden in athymic nude female mice injected with PC-9-Br cells. The majority of tumor burden is within the brain, with a smaller amount of vertebral metastases. **b-e** Micro-CT reconstruction of a mouse with a PC-9-Br tumor shows the anatomical location of the tumor. **f** Median survival for the PC-9 parental line is 61.5 days (*n* = 2), which is reduced in brain-seeking PC-9-Br line (45.5 days, *n* = 4)

In order to create a brain seeking variant of the PC-9 lung cancer cell line, PC-9 cells were injected intracardially and extracted from brain tissues, of nude mice for a total of 5 rounds using the method developed by Yoneda et al. [13]. The cells from this sixth round were “brain-seeking” (PC-9-BR), as there was very little evidence of peripheral disease after the intracardiac injection. Figure 1 shows the distribution of the sixth round of PC-9 injections (Fig. 1a), stills from a 3D reconstruction of a mouse with brain tumor (Fig. 1b-e), and the survival curve of the parental and brain-seeking PC-9 line (Fig. 1f). While the median survival was 61.5 days (*n* = 2) in the parental line, the median survival for the brain-seeking line was shorter at 45.5 days (*n* = 4).

### PC-9-Br creates numerous, widespread, and various sized brain metastases

**Fig. 2:**
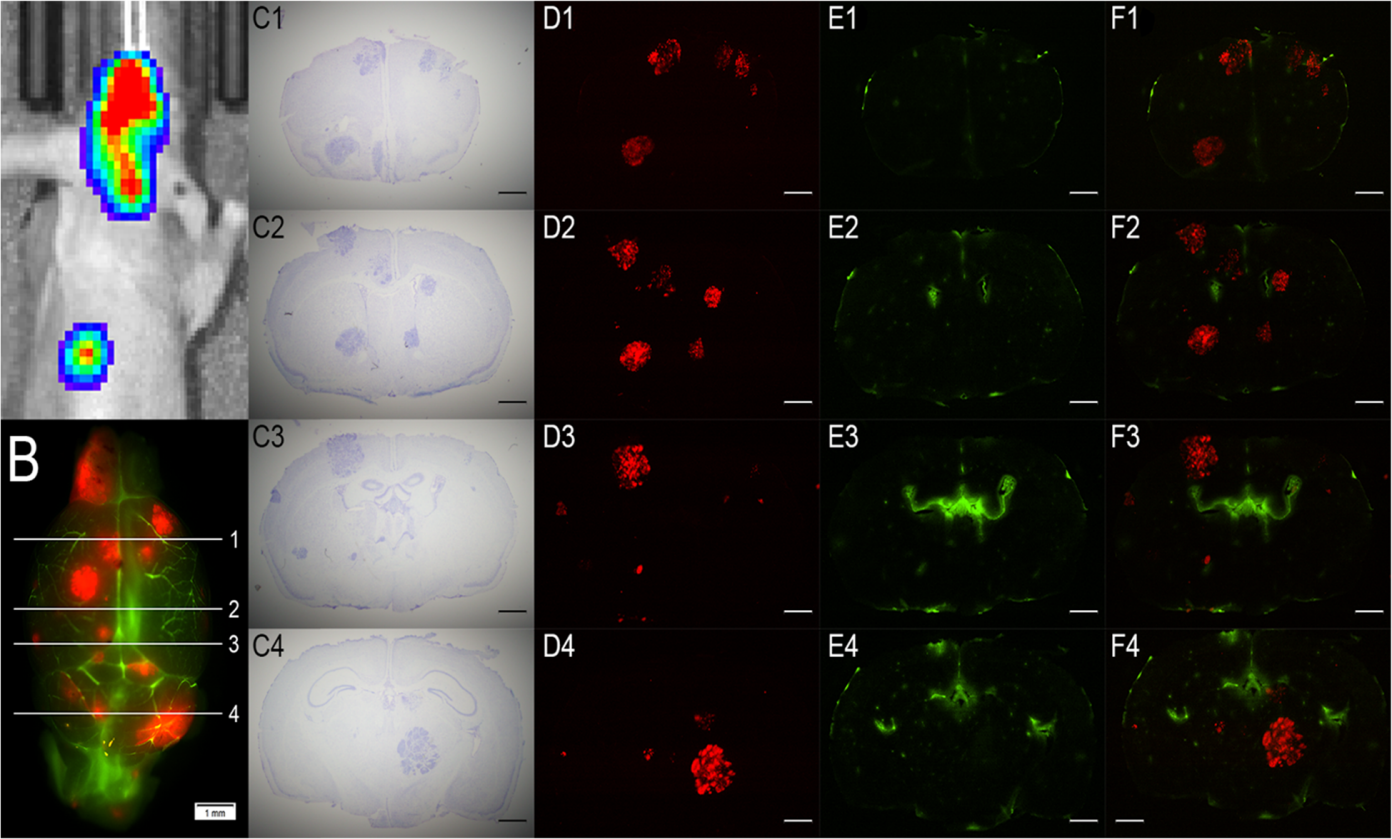
**a** Luciferin bioluminescence shows the large PC-9-Br tumor burden. **b** Fluorescence imaging contrasts the Oregon Green-perfused vasculature and the distribution of Tomato Red-expressing tumors. Four numbered slices correspond to the coronal sections (c-f). **c** Visualization of brain metastases based on cresyl violet staining. **d** Tomato Red tumors accurately represent tumor burden confirmed by cresyl violet staining. **e** Oregon Green highlights normal and disrupted vasculature in tumor brain. **f** An overlay of Oregon Green and Tomato Red depicts tumor environment and vascular integrity. Scale bars = 1 mm

PC-9-Br cells formed numerous, widespread tumors within the brain parenchyma. Figure 2 presents the metastatic lesions and cerebral vasculature from the frontal cortex to the cerebellum. Bioluminescence (Fig. 2a) and fluorescence (Fig. 2B) outline the location of tumors within the brain. Four coronal slices were taken 800–1600 μm apart, which are depicted in a brain atlas (Fig. 2 C1-F4).

### Non-targeted chemotherapy cisplatin+etoposide significantly prolonged survival of PC-9-Br brain metastases compared to vehicle control or cisplatin+ pemetrexed

**Fig. 3:**
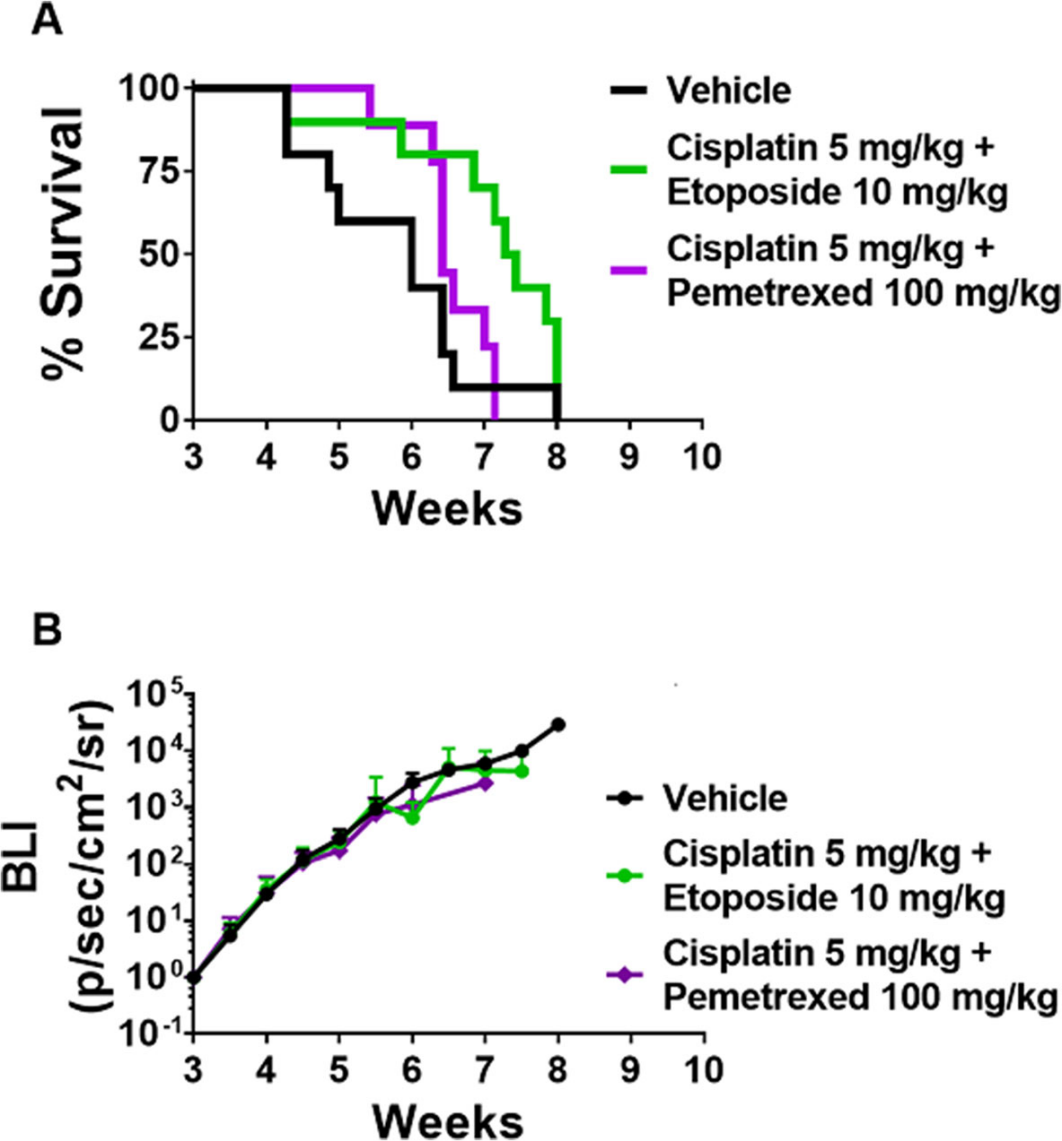
Traditional lung cancer chemotherapy fails to extend survival and limit CNS tumor burden progression. **a** On day 21 after intracardiac injection of PC-9-Br cells, mice were treated with vehicle (saline, *n* = 10), combined cisplatin+etoposide (*n* = 10), or combined cisplatin+pemetrexed (*n* = 9). Median survival time was 42 days for vehicle, 51.5 days for cisplatin+etoposide, and 45 days for cisplatin+pemetrexed. Cisplatin+etoposide significantly improved median survival compared to vehicle (*p* < 0.05), though cisplatin+pemetrexed did not (*p* > 0.05). All data was analyzed using log-rank statistics. **b** Mean BLI signal plotted versus time in mice exhibiting intracranial metastases

To evaluate the efficacy of traditional chemotherapy of physician’s choice in our preclinical model, we inoculated female athymic nude mice with the PC-9-Br cell line and treated with standard clinical agents. Mice treated with the conventional chemotherapeutic combinations cisplatin with etoposide or cisplatin with pemetrexed resulted in BLI signal maximum increases of 4400-fold and 2700-fold, respectively (Fig. 3b). Survival in the mice receiving cisplatin+etoposide was 51.5 days, which was significant longer when compared to vehicle control (42 days) (*p* < 0.05), while the mice receiving cisplatin+pemetrexed survived for 45 days, which was insignificant when compared to vehicle (Fig. 3a & Table 1). Table 1 also shows that the median size of tumors in mice receiving cisplatin+etoposide (0.1093 mm^2^) was significnalty smaller than that of cisplatin+pemetrexed (0.2492 mm^2^) or vehicle control (0.1844 mm^2^) (p < 0.05). While cisplatin+etoposide significantly increased survival compared to vehicle or cisplatin+pemetrexed, overall survival remains poor, which is consistent with clinical outcomes [8].

**Table 1:** Survival time and sizes of PC-9-Br tumors based on drug treatment

Therapy	n	Survival (days)	Median size (mm^**2**^)	IQR (mm^**2**^)
Vehicle	114	42	0.1844	0.1129–0.3097
Cisplatin+Pemetrexed	96	45^a^	0.2492^a^	0.1305–0.4054
Cisplatin+Etoposide	117	51.5^b,c^	0.1093^b,c^	0.0533–0.2384

Values bearing the letter ^a^ indicate no significant differences compared with vehicle, those labeled ^b^ denote a significant difference when compared with vehicle, and ^c^ denotes a significant difference when is cisplatin+etoposide compared with cisplatin+pemetrexed

### Cisplatin+etoposide-treated tumors have significantly higher ICG fluorescence intensity than non-tumor regions in comparision to cisplatin+pemetrexed or vehicle-treated tumors

**Fig. 4:**
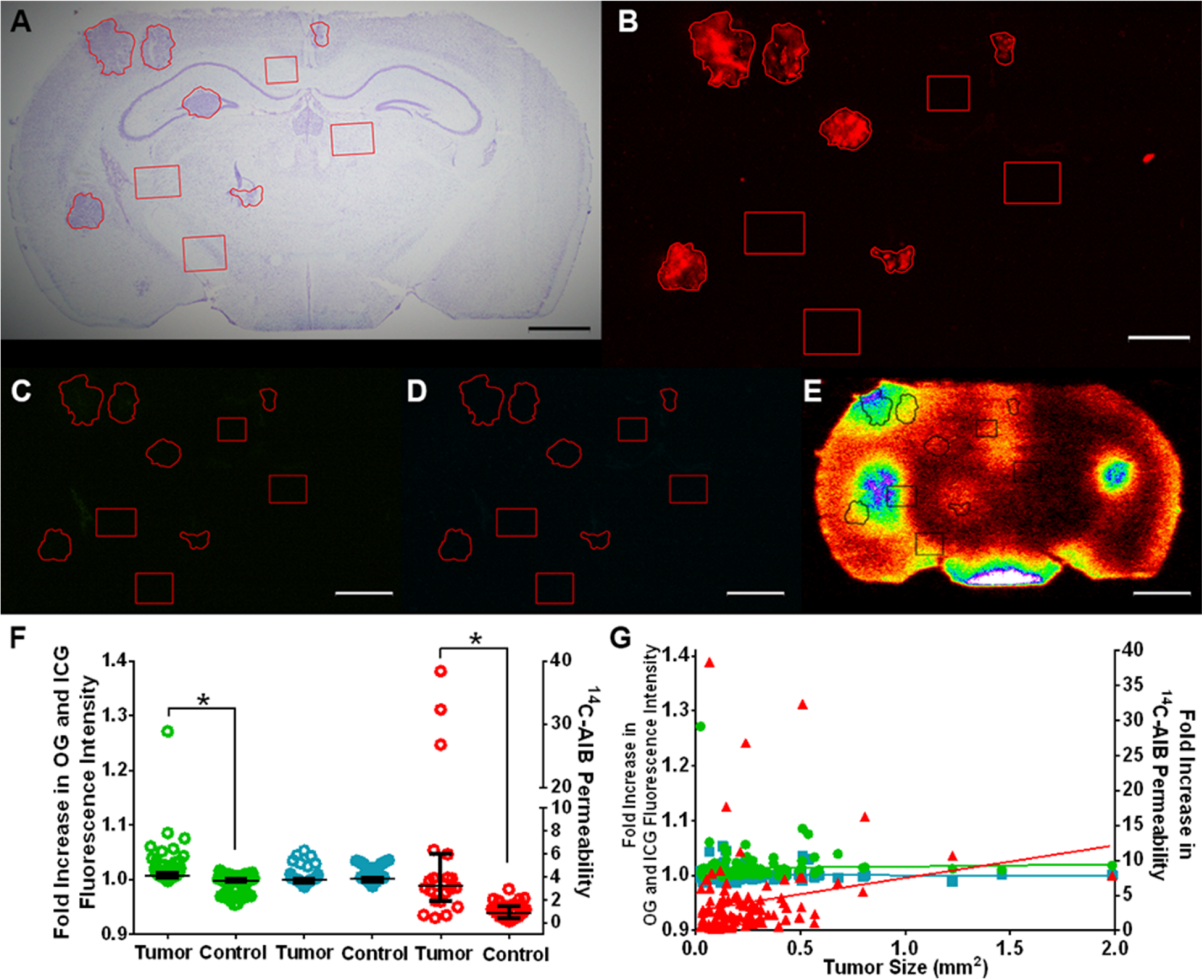
Permeability changes of PC-9-Br treated with vehicle. **a** A representative cresyl violet brain slice of vehicle-treated PC-9-Br tumors, with **b** corresponding Tomato Red tumor fluorescence. **c** The same slice with Oregon Green, **d** ICG, and **e**^14^C-AIB auto-radiographic data to quantify permeability increases. **f** The median and interquartile ranges for fold-increases of passive permeability markers in 114 tumors over control regions. For vehicle brains, tumors were significantly more permeable to OG (green) and ^14^C-AIB (red) (*p* < 0.05), but not ICG (blue) (*p* > 0.05). **g** The fold increases of OG (green), ICG (blue), or ^14^C-AIB (red) were not correlated with metastases size (*r*^2^ < 0.02). For all depicted brain slices, tumor regions are outlined while control areas are squares. Scale bar = 1 mm

**Fig. 5:**
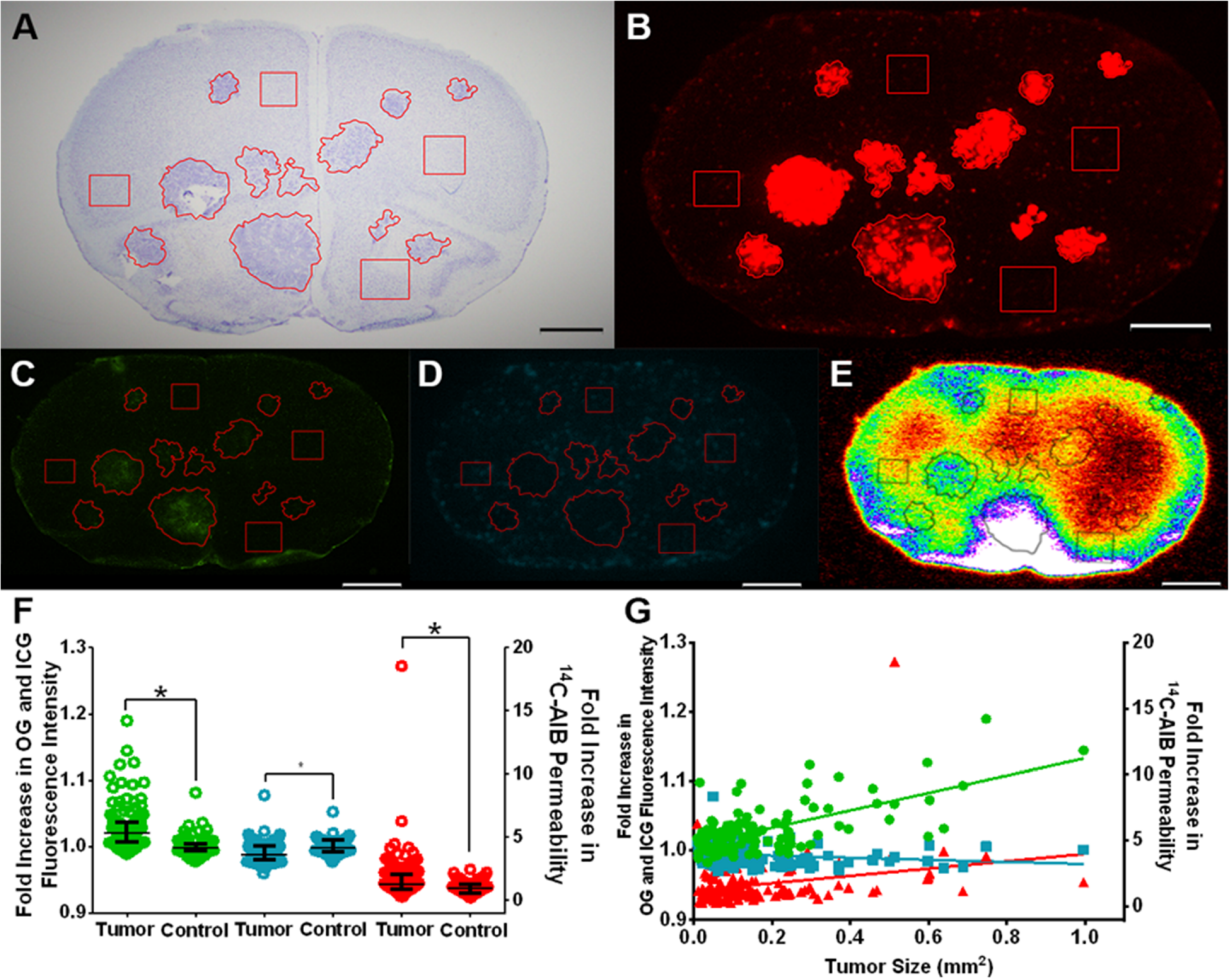
Passive permeability changes of PC-9-Br treated with cisplatin+etoposide. a A representative cresyl violet brain slice (approximately corresponding to Fig. 2 C1) of cisplatin+etoposide-treated PC-9-Br tumors, with b corresponding Tomato Red tumor fluorescence. **c** The same slice with Oregon Green, **d** ICG, and **e**^14^C-AIB autoradiographic data to quantify P-gp, vascularity, and permeability increases, respectively. **f** The median and interquartile ranges for fold-increases of dyes in 117 tumors over control regions. For cisplatin-etoposide-treated brains, tumors were significantly more permeable ^14^C-AIB (red) and OG (green) and ICG (blue) than control regions (*p* < 0.05). **g** The fold increases of OG (green), ICG (blue), or ^14^C-AIB (red) were not correlated with metastases size (*r*^2^ < 0.02). For all depicted brain slices, tumor regions are outlined while control areas are squares. Scale bar = 1 mm

**Fig. 6:**
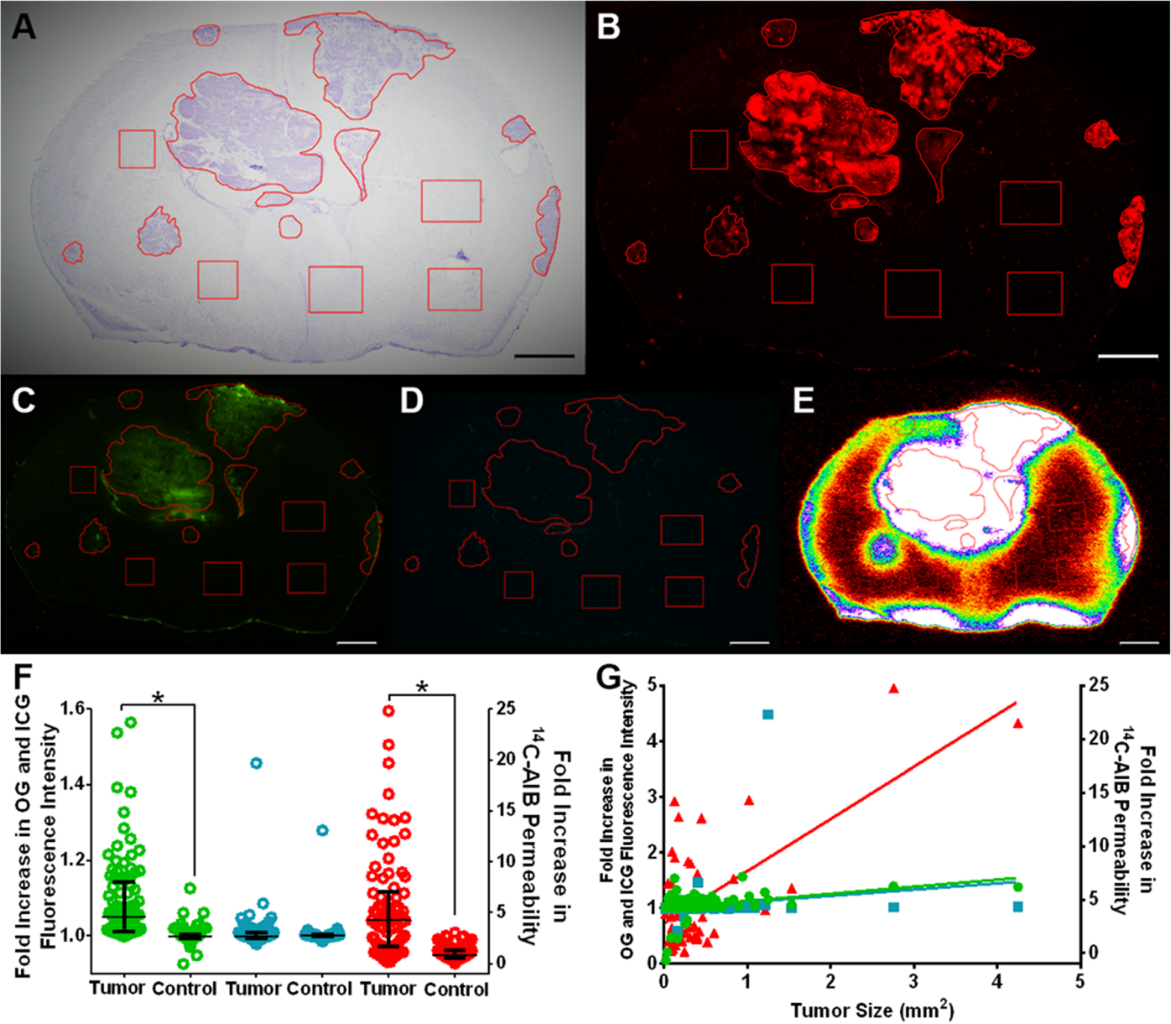
Passive permeability changes of PC-9-Br treated with cisplatin+pemetrexed. **(a)** A representative cresyl violet brain slice (approximately corresponding to Fig. 2 C3) of cisplatin+pemetrexed-treated PC-9-Br tumors, with **(b)** corresponding Tomato Red tumor fluorescence. **c** The same slice with Oregon Green, **d** ICG, and **e**^14^C-AIB autoradiographic data to quantify P-gp, vascularity, and permeability increases, respectively. **f** The median and interquartile ranges for fold-increases of passive permeability markers in 96 tumors over control regions. For cisplatin-pemetrexed-treated brains, tumors were significantly more permeable to ^14^C-AIB (red) and OG (green) (*p* < 0.05), but not ICG (blue) (*p* > 0.05). **(G)** While the OG (green) intensity had a modest correlation with mm^2^ (*r*^2^ = 0.42), ICG (blue) and ^14^C-AIB (red) were not correlated with metastases size (*r*^2^ < 0.15). For all depicted brain slices, tumor regions are outlined while control areas are squares. Scale bar = 1 mm

As animals became moribund with neurological symptoms, we sought to determine the extent and differences of passive permeability, P-gp efflux, and vascularity of control and drug-treated tumors via use of three different molecular weight markers (Figs. 4, 5 and 6). As shown in Fig. 4, passive permeability changes in vehicle metastatic lesions ranged from 0.45 to 38.39-fold over normal brain with a median (IQR) fold change of 3.25 (1.93–5.97) for ^14^C-AIB (Fig. 4f), which were significantly higher than non-tumor regions (*p* < 0.01). For OG, fluorescence intensity ranged from 0.997 to 1.271-fold with a median (IQR) fold change of 1.007 (1.004–1.013), which was significantly higher than non-tumor regions (*p* < 0.01). For ICG, fluorescence intensity ranged from 0.987 to 1.053-fold with a median (IQR) fold change of 1.0 (0.995–1.002), which was not significantly higher than non-tumor regions (*p* > 0.05). No correlation was observed (*r*^2^ < 0.02) for OG, ICG, or ^14^C-AIB passive permeability and metastasis size (Fig. 4g).

After seeing a positive trend in vehicle-treated tumors, we characterized tumors treated with conventional chemotherapy cisplatin+pemetrexed or cisplatin+etoposide. As shown in Fig. 5, passive permeability changes in cisplatin+etoposide metastatic lesions ranged from 0.30 to 18.55-fold over normal brain with a median (IQR) fold change of 1.23 (0.854–2.077) for ^14^C-AIB (Fig. 5f), which was significantly higher than non-tumor regions (*p* < 0.01). For OG, fluorescence intensity ranged from 0.989 to 1.190-fold with a median (IQR) fold change of 1.020 (1.007–1.037), which was significantly higher than non-tumor regions (*p* < 0.01). For ICG, fluorescence intensity ranged from 0.960 to 1.078-fold with a median (IQR) fold change of 0.989 (0.981–1.001), which was significantly higher than non-tumor regions (*p* > 0.01). There was a no correlation (*r*^2^ = 0.07) to changes in ^14^C-AIB permeability and lesion size, while a moderate correlation was observed (*r*^2^ = 0.42) for OG but not ICG (*r*^2^ = 0.03) fluorescence intensity and metastasis size in the cisplatin-etoposide model **(**Fig. 5g).

As shown in Fig. 6, passive permeability changes in cisplatin+pemetrexed brain tumors ranged from 0.160 to 24.83-fold over normal brain with a median (IQR) fold change of 4.235 (1.681–7.046) for ^14^C-AIB (Fig. 6f), which was significantly higher than non-tumor regions (*p* < 0.01). For OG, fluorescence intensity ranged from 0.065 to 1.565-fold with a median (IQR) fold change of 1.049 (1.010–1.144), which was significantly higher than non-tumor regions (*p* < 0.01). For ICG, fluorescence intensity ranged from 0.593 to 4.490-fold with a median (IQR) fold change of 0.999 (0.994–1.005), which was not significantly higher than non-tumor regions (*p* > 0.05). There was a moderate correlation (*r*^2^ = 0.44) in ^14^C-AIB permeability and lesion size. No correlation was observed for OG (*r*^2^ = 0.12) or ICG (*r*^2^ = 0.03) fluorescence intensity and metastasis size in the cisplatin-pemetrexed model (Fig. 6g).

### The PC-9-Br developed significant acquired resistance to gefitinib in vitro compared with PC-9 parental and its potential molecular mechanisms

**Fig. 7:**
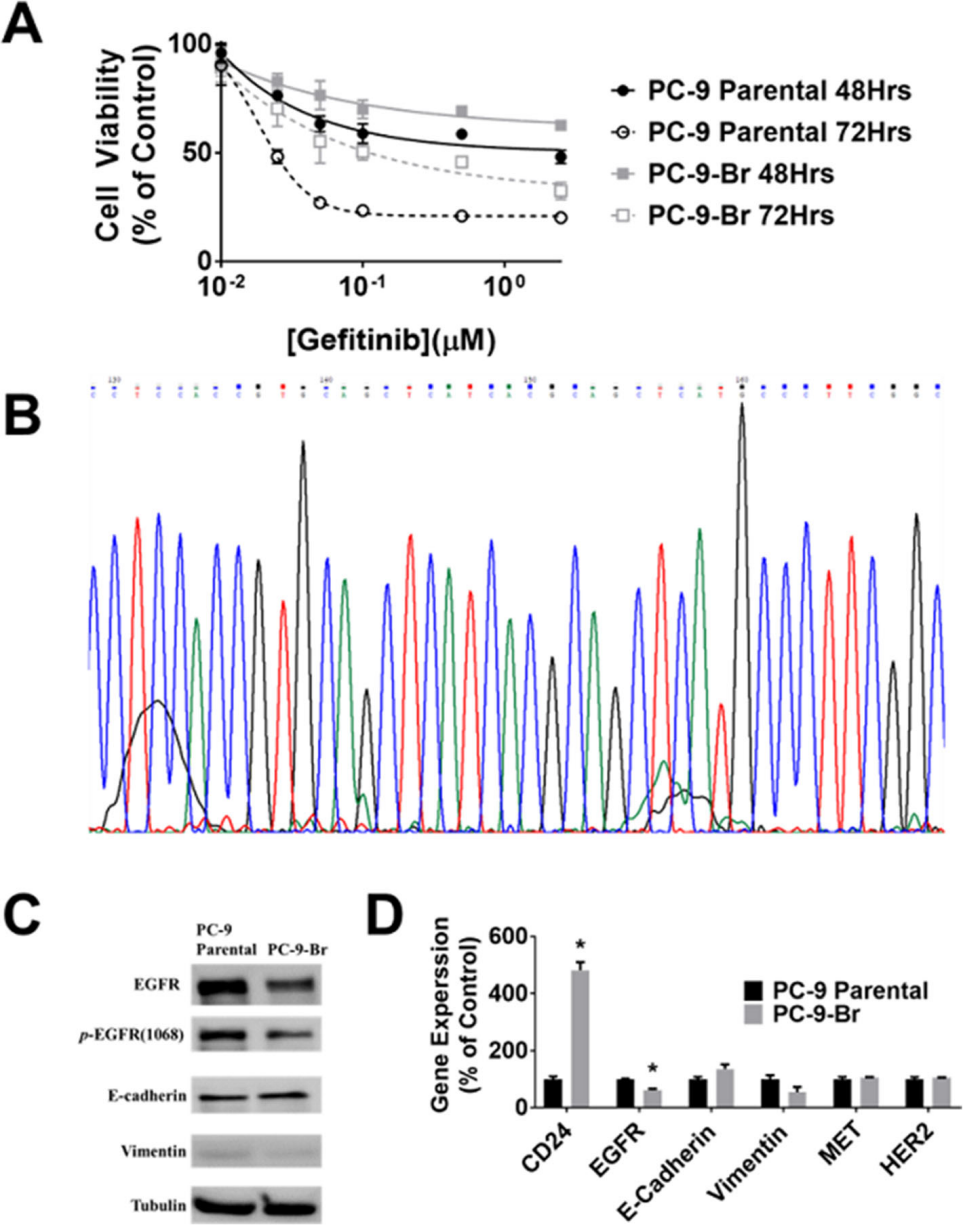
In vitro characterization of PC-9 Parental and PC-9-Br. **a** Cytotoxic effects of gefitinib on PC-9-Br and PC-9 parental at 48 h and 72 h. Data are expressed as the percentage by comparing vehicle control determined by the MTT assay. Values are represented as mean ± SD, *n* = 6. **b** Gene analyses of PC-9-Br showing no T790M (c.2369C > T) was found in EGFR exon 20 of PC-9-Br. **c** Western blot analyses of EGFR, *p*-EGFR (Y1068), and EMT biomarkers (E-cadherin and Vimentin) in PC-9 parental and PC-9-Br. **d** qRT-PCR analyses of PC-9-Br compared to PC-9 parental. Data are mean ± SD. “*” indicates a significant difference between PC-9-Br and PC-9 parental analyzed by a Student’s t test (*p* < 0.05). Full western blot images are presented in **supplementary figure****1**. The Bio-Rad ChemiDoc™ imaging system was used for western blot image acquisition

Figure 7a shows that the IC_50_s of gefitinib in PC-9 parental at 48 h and 72 h were 0.75 and 0.027 μM, respectively. On the other hand, the IC_50_s of PC-9-Br at 48 h and 72 h were > 2.5 and 0.1 μM, respectively. These results indicated that PC-9-Br became resistant to gefitinib in comparison with PC-9 parental in vitro. DNA sequencing showed the same EGFR mutational spectrum in the analyzed EGFR exon 20 in PC-9-Br compared to PC-9 parental, in which no T790M was detected (Fig. 7b). No significant changes of E-cadherin and vimentin, important markers of epithelial mesenchymal transition (EMT), were observed in PC-9-Br compared with PC-9 parental by analyses of both Western blot (Fig. 7c) and qRT-PCR (Fig. 7d). The protein expressions of EGFR and *p*-EGFR (1068) were significantly downregulated in PC-9-Br compared with PC-9 parental (Fig. 7c). The decreased gene expression of EGFR was confirmed by the result of qRT-PCR (Fig. 7d). Meanwhile, it was found that the markers of cancer stem cells (CSC) CD24 was significantly increased and no MET and HER amplications were detected in PC-9-Br compared to PC-9 parental (Fig. 7d). These data suggested that loss of EGFR and *p*-EGFR might contribute to gefitinib resistance of PC-9-Br compared with PC-9 parental.

## Discussion

The aim of the current study is to explore the causal relationship between LCBM and drug resistance, though previous studies mostly reported the metastasis of lung cancer induced by acquired drug resistance [17, 18]. The PC-9 cell line bearing the EGFR del 19 mutation sensitive to EGFR-TKIs was developed into brain-seeking metastatic lines (PC-9-Br) and studied in vivo in the context of chemotherapeutic efficacy, where PC-9-Br showed resistance to non-targeted chemotherapy. The in vitro resistance to EGFR-TKI gefitinib was also found in PC-9-Br which showed loss of EGFR and *p*-EGFR expressions as resistant mechanisms. The results of our study may provide new insights into development of therapeutic strategies for treating NSCLC with drug resistance induced by brain metastasis.

To create a LCBM model, two main methods exist: intracardiac and intracarotid injections [19]. While intracarotid injections deliver cancer cells directly to the brain compared to intracardiac injections which allow cancer cells to circulate throughout the arterial system, intracarotid injections are much more invasive and time-consuming, and often have similar results to intracardiac injections, though there is concern of regionally induced stroke like symptoms with intracarotid injections [20].

The PC-9-Br line expresses the efflux pump P-gp [21]. Herein, we use the passive permeability marker ^14^C-AIB, a P-gp substrate OG, and vascular density marker ICG to study effects of chemotherapy on tumor vasculature in the PC-9 model of LCBM. Permeability of these markers was studied in brains treated with vehicles, cisplatin+etoposide, or cisplatin+pemetrexed. We observed that the PC-9-Br was more resistant to chemotherapy than the parental counterpart (PC-9 parental). The passive permeability of ^14^C-AIB was generally significantly higher in tumor regions compared to non-tumor regions. In contrast, there was no significant correlation between tumor size and ^14^C-AIB permeability. PC-9-Br tumors are generally less than 1 mm^2^ and far less permeable to both ^14^C-AIB and similarly-sized fluorescent markers [22]. This is in contrast with primary glioblastoma, whose lesions are much larger and much more permeable to ^14^C-AIB, with rates of transfer that near water diffusion [23]. OG and ICG fold increases varied between each treatment group and were not predictable. Tumor sizes are smaller in treatment groups that extend median survival. Lastly, we observed that there was no correlation between survival and tumor size (data not shown). This is the first paper to illustrate the heterogeneity of tumor distribution and vascular permeability of lung-brain metastases, especially in the context of therapeutic treatment.

The resistance of LCBM to chemotherapy is mainly due to the physiochemical activities of the BBB and BTB [24, 25]. The physical BBB is composed of endothelial cells joined by tight junctions, a basement membrane, pericytes, and astrocytic foot processes [26]. Efflux transporters such as P-gp, breast cancer resistance protein (BCRP), and intracellular enzymes (phosphatases, oxidases) comprise the chemical portion of the BBB, further restricting brain penetration of chemotherapy [26, 27]. In brain metastases, vasculature is often compromised, resulting in the BTB. Though often described as “leaky”, vascular disruption in the BTB does not always significantly impact chemotherapeutic penetrance [9, 11].

In our in vivo study, it was observed that ICG fluoresence intensity in cisplatin+etoposide treated tumors was higher than in non-tumor regions. In contrast, no higher ICG fluoresence intensity was observed in vehicle or cisplatin+pemetrexed treated tumors compared with non-tumor regions. These results indicated that brain vascular density and surface area surounding the brain tumors were higher in cisplatin+etoposide treatment groups than in vehicle or cisplatin+pemetrexed treatment groups. This suggests the potential increases in angiogenesis and drug delivery in the cisplatin+etoposide group, which may correlate with the increased survival observed in the study. However, platinum-based therapy, including cisplatin+etoposide and cisplatin+pemetrexed, have shown limited efficacy in multiple Phase II trials involving EGFR-mutated LCBM [8]. Platinum doublet therapy has largely been replaced by the use of targeted inhibitors. While platinum combinatorial approaches are being phased out, it is still important to show that our preclinical model also follows the trend of targeted therapy superiority.

PC-9 is commonly utilized in preclinical lung cancer research to evaluate the effects of chemotherapy in an EGFR-mutant model [28–30]. PC-9 cells are also sensitive to first generation (gefitinib and erlotinib) and second generation (afatinib) tyrosine kinase inhibitors, and can be induced to form the T790M mutation which often leads to drug resistance and relapse in the clinical setting [31, 32]. While the PC-9 is commonly used for preclinical research, the PC-9-Br cell population presents a brain specific variant, providing a scenario in which targeted treatment strategies can be efficiently tested for brain metastases of lung cancer.

Despite being substrates for P-gp efflux, it was demonstrated that erlotinib [33] and gefitinib [34] enter brain metastatic parenchyma, and numerous case reports show prolonged survival and positive outcomes using these first-line EGFR-tyrosine kinase inhibitors [35, 36]. Gefitinib has been shown to be superior to carboplatin-pemetrexed therapy in prolonging progression-free survival in EGFR-mutated brain metastases [37]. However, in our in vitro study, PC-9-Br showed significant resistance to gefitinib. Further molecular mechanism study revealed neither a T790M mutation nor amplications of MET and HER2, as typical resistant mechanisms, were found in PC-9-Br compared with parental PC-9. On the other hand, significant losses of EGFR and *p*-EGFR were detected in PC-9-Br compared to parental PC-9, which was also reported in other NSCLC EGFR-mutant cell lines as one EGFR-TKI resistant mechanim [38, 39]. The significantly increased gene expression of CD24, as an important marker of cancer stem cells (CSCs), was detected in PC-9-Br compared to PC-9 parental, which may be another metastatic mechanism in this LCMB model. It is also interesting to note that in our study EMT, as a very common mechanism of cancer cell invasion and tumor metastasis, was not found in PC-9-Br compared with PC-9 parental. It may signify that other mechasims may exist underlying the lethal LCBM as observed in our study, which will be investigated in future studies.

## Conclusion

The EGFR-mutant PC-9-Br creates many scattered brain metastases, most of which are smaller than 1.0 mm^2^. These tumors had an active P-glycoprotein efflux mechanism. Conventional chemotherapy such as cisplatin and pemetrexed were not as effective in increasing median survival as cisplatin and etoposide, but tumors treated with cisplatin+etoposide have smaller tumor sizes and lower ^14^C-AIB permeability, despite increased vascular density. Fluorescence microscopy revealed more vascular formations in tumors compared to non-tumor regions in cisplatin+etoposide treated group, which was not observed in cisplatin+pemetrexed treated or vehicle control group. This difference may be correlated with more effectiveness of cisplatin+etoposide treatments on prolonging the survival time of LCBM mice compared to cisplatin+pemetrexed treatment or vehicle control. This model for LCBM may prove useful for improving translational research. More importantly, PC-9-Br exhibited more resistance to gefitinib treatment compared with PC-9 parental in vitro. Further studies on molecular mechanisms revealed that the gefitinib drug resistance in PC-9-Br might result from loss of EGFR and *p*-EGFR in PC-9-Br compared with PC-9 parental, instead of a T790M mutation or HER2/MET amplifications. There was no EMT found in PC-9-Br compared to PC-9 parental, suggesting the existence of other mechanisms responsible for LCBM that warrant further investigations.

## Supplementary information

**Additional file 1.** Full western blot images.

## Abbreviations

PC-9-Br: PC-9 brain seeking cell line
IC50: Half maximal inhibitory concentration
EGFR: Epidermal growth factor receptor
p-EGFR: Phosphorylated epidermal growth factor receptor.
EMT: Epithelial-mesenchymal transition
MET: Hepatocyte growth factor receptor
HER2: Human epidermal growth factor receptor 2
EGFR-TKI: Epidermal growth factor receptor tyrosine kinase inhibitor
LCBM: Lung cancer brain metastasis
KRAS: Kirsten rat sarcoma viral oncogene
EML4-ALK: Echinoderm microtubule-associated protein-like 4 fused to anaplastic lymphoma kinase
NSCLC: Non-small cell lung carcinoma
SCLC: Small cell lung carcinoma
BBB: Blood-brain barrier
BTB: Blood-tumor barrier
14C-AIB: 14C-aminoisobutyric acid
P-gp: P-glycoprotein
OG: Oregon Green
ICG: Indocyanine green
BLI: Bioluminescence
ROI: Region of interest
CSC: Cancer stem cells
BCRP: Breast cancer resistance protein
CNS: Central nervous system
