# Supplementary figures and images for: Drug resistance occurred in a newly characterized preclinical model of lung cancer brain metastasis
Source: BMC Cancer. 2020 Apr 7;20:292. doi: 10.1186/s12885-020-06808-2 (PMC7137432; doi:10.1186/s12885-020-06808-2)

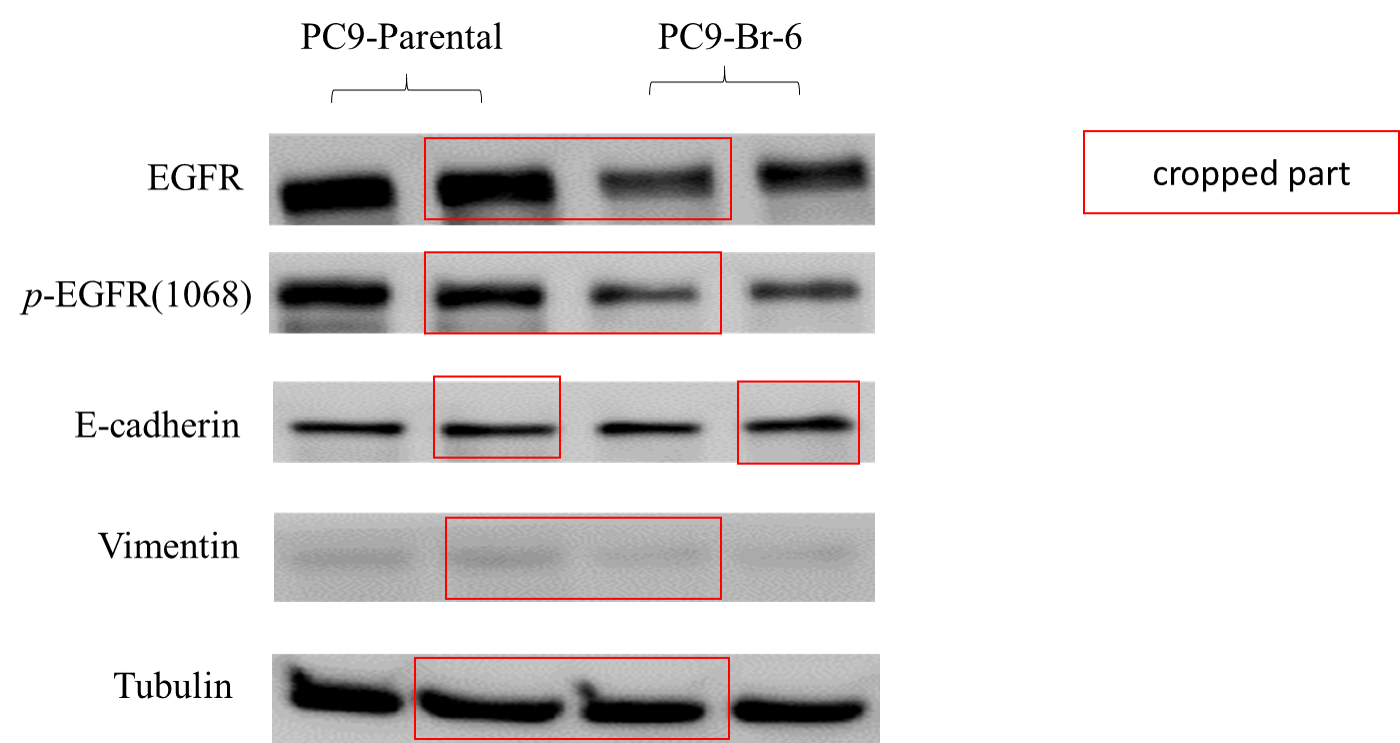

Supplement: Supplementary file 1 — Additional file 1. Full western blot images. [file 12885_2020_6808_MOESM1_ESM.tif]
